# Willingness to maintain contracts with family doctors among Chinese residents: results from one national cross-sectional study and a meta-analysis of 25 studies

**DOI:** 10.3389/fpubh.2023.1162824

**Published:** 2023-12-22

**Authors:** Xinyan Li, Jun Ye, Jing Feng, Qiaosen Chen, Ge Qu, Zhengyi Wan, Zihui Lei, Adamm Ferrier, Heng Jiang, Yanling Zheng, Yong Gan

**Affiliations:** ^1^Department of Social Medicine and Health Management, School of Public Health, Tongji Medical College, Huazhong University of Science and Technology, Wuhan, Hubei, China; ^2^Department of Public Management, School of Public Health and Management, Wenzhou Medical University, Wenzhou, Zhejiang, China; ^3^Division of Cardiovascular Medicine, Department of Medicine, Karolinska Institutet, Huddinge, Stockholm, Sweden; ^4^Department of Public Health, School of Psychology and Public Health, La Trobe University, Melbourne, VIC, Australia; ^5^Melbourne School of Population and Global Health, University of Melbourne, Melbourne, VIC, Australia; ^6^Department of General Practice, Shouyilu Street Community Health Service Center, Wuhan, Hubei, China

**Keywords:** family doctor, contract services, renewal willingness, meta-analysis, cross-sectional studies

## Abstract

**Background:**

A number of studies have investigated the influencing factors regarding the renewal of contracts associated with Family Doctor Contract Services (FDCS) in different regions of China since it was officially implemented in 2009; however, none of the previous studies have been considered using a nationally representative sample in combination with a meta-analysis.

**Methods:**

A multistage stratified sampling method was used to investigate participants’ socio-demographic characteristics, health status, understanding, use, and evaluation of the FDCS, and their willingness to renew contracts in Eastern, Central, and Western China from September to November 2021. We searched the PubMed, Ovid Medline, CNKI, VIP, Wanfang, and SinoMed databases to retrieve previous studies related to the willingness of Chinese residents to renew contracts with their family doctor (FD), and a meta-analysis was performed to systematically summarize the willingness to maintain contracts and influencing factors.

**Results:**

Among 2,394 residents, 2,122 (88.64%) were willing to renew their contracts. The mixed-effect logistic regression model results demonstrated that residents who (1) preferred primary health service institutions, (2) had a better knowledge of FDCS, (3) were more willing to visit primary health service after signing the contract with FDs, (4) were not intending to change FDs, (5) were satisfied with FDCS, and (6) trusted in FDs reported a higher level of willingness to maintain contracts with FDs. Our meta-analysis confirmed that older age, being married, having chronic diseases, choosing primary medical institutions for the first contact, having a good knowledge of FDCS/FDs, being satisfied with FDCS and the medical skills of FDs, and trusting FDs were all positively associated with residents’ willingness to renew contracts (*p* < 0.05).

**Conclusion:**

The willingness of consumers to maintain contracts with FDs in China varies in different areas. Giving priority services to groups of high need contributed to an improved rate of renewal. We suggest that in order to continue to increase annual contract renewal, it is necessary to strengthen consumer awareness through effective marketing and continue to work toward meeting consumer expectations, thereby increasing confidence and trust in FDCS.

## Background

The 2018 Global Conference on Primary Health Care in Astana, Kazakhstan, highlighted that promoting and enhancing the primary healthcare system is a critical global priority in the following decade (1). Family doctors (FDs), often referred to as the gatekeepers of residents’ health, are the backbone of the primary healthcare system (2). Indeed, effective deployment of primary health can have system-wide benefits by ensuring that patients are directed to the appropriate secondary and tertiary health services. The efficiency, effectiveness, and quality of a country’s healthcare system are therefore directly associated with the quality and availability of the general practitioner (GP) workforce. Consumer access to primary health provided by general practitioners varies across the world: in the United Kingdom and New Zealand, one must be registered to a specific GP for any treatment other than emergencies (3), whereas in other countries (such as Australia, Canada, China, France, and the USA), a consumer is free to select a primary health provider of their choice and at will (4), and often develops a preference for a continued doctor–patient relationship. Satisfaction with primary health service provision is challenging, and Comino et al. note that “services are not always readily available, accessible, or affordable” (5).

China’s Family Doctor Contract Service (FDCS) is a relatively new model of primary healthcare provision within community health services. By signing two-way voluntary healthcare service contracts with community residents, FDs provide a mechanism that contributes to comprehensive, continuous, and accessible personalized community health services to the contracted residents according to local conditions, and gradually guide the establishment of the System of First Treatment in Community, Two-way Referral and Hierarchic Healthcare (6). FDCS has been a crucial part of comprehensive, prevention-oriented, and people-centered healthcare in China.

The government of China acknowledged the importance of primary healthcare and family doctors (or as they are often termed in the West “primary healthcare physicians”) with a strategic focus on human resource development, particularly in areas of preparatory and ongoing training, utilization, and motivation. In 2018, the General Office of the State Council of the People’s Republic of China announced further programs to reform and improve the training, utilization, and motivation of FDs. The instructions pointed out that by the year 2020, the occupational attractiveness to medical graduates of the primary healthcare role should have improved (7). Later the same year, the National Health Commission issued policies to further standardize the service provision and expectations of FDCS (8), in order to standardize consumer service quality expectations and demands with a view to expanding the contract uptake. In 2021, the 14th Five-Year Plan (2021–2025) for National Economic and Social Development and the Long-Range Objectives Through the Year 2035 identified the need to (1) strengthen and encourage further development of grassroots medical teams, (2) steadily expand the coverage of FDCS in both rural and urban areas, and (3) improve the quality of contract services (9).

The FDCS was launched in China in 2009 as an innovative and fundamental policy in New Medical Reform (10). Since then, various provinces and cities successively launched and explored different models of FDCS. For example, the “1 + 1 + 1” model in Nanning (11), where the residents voluntarily choose one secondary and one tertiary hospital as a referral hospital at the same time as signing a contract with the family doctor, and the “Co-management of Doctors of Three Kinds” model in Xiamen (12), which was that residents signed contracts with a team composed of a specialist from the tertiary hospital, a GP from the primary care institution, a certified health manager. Regardless of the varied interpretations of FDCS in different regions, priority was always given to key groups such as the poor, the older adults, the disabled, pregnant women, children, and patients with chronic diseases. The FDCS aimed to provide residents with comprehensive healthcare, allowing most residents, especially those with chronic conditions, to manage their health needs at primary care institutions. By the end of 2018, there were 382,000 contracted FD teams in China, serving 320 million people (71.3%) of target groups (13).

In China, the contract service cycle is nominally for 1 year, and residents could either voluntarily renew or terminate the contract and select a new provider at the end of the year. Previous studies have investigated the quality and effect of FDCS mainly focusing on the aspects of residents’ awareness of FDs (14–17), willingness to sign a contract (17–20), trust (21–25), satisfaction (17, 18, 21–24, 26–28), and so on. Nevertheless, studies have shown that there were limitations in these evaluation indices in terms of their comprehensiveness, pertinence, and practice guidance (29). Importantly, in comparison with the initial uptake rate of FDCS, the likelihood of subsequent contract renewal was considered a strong and important indicator of value (21), as it was suggested as a proxy for consumer satisfaction of the FDCS model in maintaining consumer interest and awareness of personal long-term benefits, quality, and effectiveness of care provided.

To date, some studies have investigated the likelihood of contract renewal by consumers and the influencing factors, which were limited to one provincial or municipal area. In addition, no systematic review and meta-analysis had yet been conducted to summarize the evidence associated with the intention of contract renewal and the influencing factors by Chinese health consumers. It was for these reasons we chose to address these important research gaps. The results of this study could contribute to the establishment of effective strategies and policies to promote the high-quality development of FDCS and provide comparative valuable evidence for family practice research internationally.

## Methods

### Study design and settings

A cross-sectional survey was designed and conducted in six major cities across China from September to November 2021. Using a multistage stratified random method, we selected six cities from provinces of Eastern China (Suzhou, Wenzhou), Central China (Wuhan, Changsha), and Western China (Nanning, Chongqing). Within each of these cities, 5–10 communities or villages (towns) were randomly selected, and in each community or village (town) 100–120 residents were randomly invited to complete an anonymous, self-administered questionnaire via WeChat.

Participation in the study was strictly on a voluntary basis, and written informed consent was obtained from each participant. Participant information was treated confidentially: we kept consent information separate from the respondent data. Formal ethics oversight was provided by the Ethics Committee of the Wenzhou Medical University Institutional Review Board, Wenzhou, China (no. 2021–019).

### Sample and data collection

The required sample size was calculated according to the following formula: *n* = [Z^2^ π(1-π)]/ δ^2^ (n = sample size, *Z* = confidence level for a normal distribution, π = expected prevalence, and δ = absolute error). According to the previous meta-analysis, the contract rate was 46.2% (30). Taking a confidence interval (CI) of 95% and an absolute error of 1.5%, we determined an ideal sample size of 4,244. This sample size was increased to 4,700 to compensate for a potential nonresponses rate of 10%. Participants aged 18 or above and able to complete the questionnaire independently were included in the study. Those with reading problems or hospital-diagnosed psychiatric disorders were excluded from participating.

The questionnaire covered three main aspects: socio-demographic characteristics, health conditions, and the knowledge, utilization, and evaluation of provided health services. Consumer satisfaction with FDCS was measured based on a questionnaire developed by He (31), composed of 10 items, using a standard Likert score arrangement ranging from 1 (very dissatisfied) to 5 (very satisfied). Responses with a total score between 30 and 50 were regarded as satisfied, and those with 29 or less were considered dissatisfied. In this study, the *Cronbach’s α* was 0.98.

Consumers’ trust in their current contracted FDs was measured using the Chinese version of the Wake Forest Physician Trust Scale (WFPT), which was developed by Halls et al. (32) and modified by Dong et al. (33). Once again, this section comprised 10 items, each of which used a Likert score from 1 to 5; higher scores representing a higher level of trust. Residents with a score of 30 or higher were considered as trusting in their FDs, a score lower than 29 was regarded as lacking confidence in the provision of service. In this study, the *Cronbach’s α* was 0.75. Consumers’ willingness to renew their annual contract with FDs was evaluated by a single binary question, *“Whether you wanted to renew your annual contract with FDs?,”* taking “*yes*” as being willing to renew.

### Statistical analysis

We used the Statistical Package for Social Sciences (SPSS, Inc., Chicago, IL, Version 27.0) to input the information extracted from the questionnaire gathered through WeChat. Stata 16.0 (StataCorp. 2019. *Stata Statistical Software: Release 16.* College Station, TX: StataCorp LLC.) was used to conduct the statistical analysis. We represented the distribution characteristics as the number of observations with percentage (%)and analyzed the difference in the willingness of residents with different characteristics to renew their contracts with FDs by the Chi-square (*χ*^2^) test, where the significance level was accepted as a *p-*value of <0.05 (two-sided). Due to the disparities in all covariates between the three geographical regions (see Supplementary file S1), the data featured hierarchy. A mixed-effect logistic regression was applied with a random cluster effect (geographical regions) to investigate adjusted ORs (95% CI) of factors influencing consumer willingness to renew contracts. The data aggregation was assessed using the estimated interclass correlation coefficient (ICC). The significance test was two-sided and a value of *p* < 0.05 was considered statistically significant.

### Systematic review and meta-analysis

A meta-analysis provided a quantitative assessment of the willingness to renew contracts with FDs across China in addition to the influencing factors. The Preferred Reporting Items for Systematic and Meta-Analysis (PRISMA) 2020 statement was used as a basis for conducting and reporting this systematic review. Six databases were searched from their inception to 21 September 2022: PubMed, Ovid Medline, China National Knowledge Infrastructure (CNKI) Database, VIP Chinese Science and Technology Periodical Database, Wanfang Database, and SinoMed. The search strategy was developed and adjusted for each database with a combination of Mesh words, title words, keywords, and abstract words. We used “family physician*” or “general physician*” or “family doctor*” or “general practitioner*” or “contract service*” and “renew*” or “maintain*” or “continu*” or “exten*” and “China” or “Chinses” as the relevant search terms (see Supplementary file S2).

Studies were screened independently by two reviewers and eligible studies were selected according to the criteria described as follows: (a) the study was of a cross-sectional or cohort study design; (b) the study identified contract renewal rates of FDCS of residents in China; and (c) the study population was residents aged 18 years or above. We excluded studies if (a) the study included medical staff as study subjects; (b) the study did not report the resident renewal rate or the logistic regression results of various possible influencing factors on the resident renewal rate; (c) and the study reported response rate < 70%; (d) the study was a letter, a comment, a pilot study, a conference abstract, news, or a qualitative study. Finally, we included higher-quality publications when different studies used the same survey data.

Quality assessment and data abstraction of eligible studies were achieved by two researchers using an 11-point scoring system recommended by the Agency for Healthcare Research and Quality (34). Literature quality scores ranged from 0 to 11; a higher score represented higher study quality. We then extracted the following information from each study: author, year of publication, region, sample size, number of residents willing to renew the contract, effective response rate, sampling method, ORs (95% CIs), and covariates in the model.

We used a random-effect meta-analysis to estimate consumer willingness to renew contracts with FDs. To identify this, pooled ORs for potential influencing factors were calculated with a random-effect model. Subgroup analyses included stratification by publication year, study location, quality score, etc. to investigate potential sources of heterogeneity between subgroups. Subgroup differences were tested by meta-regression analysis.

*I*^2^ statistic was applied to assess heterogeneity across studies. Potential bias due to small studies was assessed using Egger’s test and was visualized using a funnel plot. The meta-analysis was conducted with Stata V.16.0. All tests were two-sided with a significance level of 0.05.

## Results

**Figure 1:**
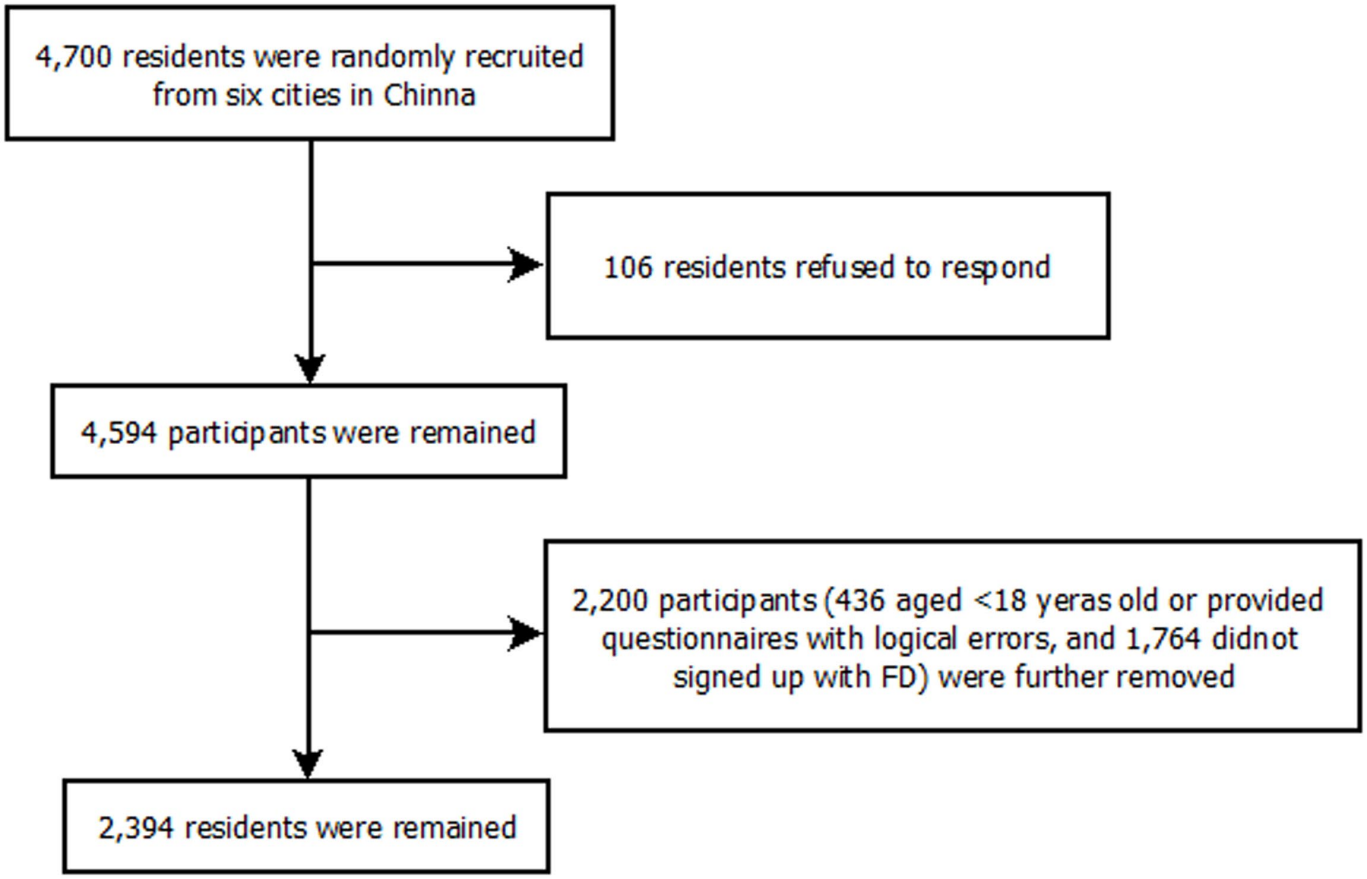
The flow chart for the sampling in this study: 6 cities, China, September to December 2021.

A total of 4,594 residents were randomly recruited in the survey with a response rate of 97.74% (4,594/4,700). As having contracted with an FD was the prerequisite for renewing a contract, after removing participants who did not meet the inclusion criteria (436 residents), finally, 2,394 eligible residents who had signed up with FDs were included in the study (see Figure 1).

Among 2,394 residents, 2,122 (88.64%) were willing to renew their contracts, and 272 (11.36%) were unwilling to renew their existing contracts. Participants aged between 18 and 97 (*M* = 49.21 years old, *SD* = 15.82). There were 1493 females, 1.66 times as many as males (n = 901), and 24.3, 42.3, and 33.4% of the respondents from Eastern (*n* = 581), Central (*n* = 1013), and Western (*n* = 800) China, respectively. The majority of participants were married (86.80%), lived in urban areas (70.55%), and reported a personal monthly income of 3001 ~ 6000 yuan (47.87%). Most (92.44%) of the respondents self-identified as having a good health status, though the prevalence of chronic diseases was 38.47%. Almost all participants had medical insurance (99.54%).

The results of the univariate Chi-square analysis suggest that respondents aged 45 ~ 64 years, from Central and Western China, who were married, who were less educated, had chronic diseases, had a walking distance to the nearest healthcare center of less than 15 min, with the preference of a primary health service for the initial consultation, who visited the primary health service institution more than 3 times in the past 12 months, who possessed a good understanding of FDCS and the services provided by the contracted FD matching their real medical needs, who were more willing to go to the primary health service in any case of illness after signing the contract with FDs, who were not intended to switch FDs, and who were satisfied with FDCS and trusted FDs have a significantly higher willingness to renew contracts with FDs (*p* < 0.05) (Table 1).

**Table 1:** Descriptive statistics and univariate analysis of the differences in willingness to renew contracts among residents.

Variables	Total (%)	Willing (%)	Unwilling (%)	*χ* ^2^	*P*
Total	2394 (100.0)	2122 (88.64)	272 (11.36)		
Age (years old)					
18 ~ 44	1024 (42.77)	877 (85.64)	147 (14.36)	16.743	<0.001
45 ~ 64	835 (34.88)	764 (91.50)	71 (8.50)		
≥ 65	535 (22.35)	481 (89.91)	54 (10.09)		
Gender					
Male	901 (37.64)	808 (89.68)	93 (10.32)	1.551	0.213
Female	1493 (62.36)	1314 (88.01)	179 (11.99)		
Geographical region					
Eastern	581 (24.27)	485 (83.48)	96 (16.52)	26.225	< 0.001
Central	1013 (42.31)	931 (91.91)	82 (8.09)		
Western	800 (33.42)	706 (88.25)	94 (11.75)		
Marital status					
Unmarried/divorced/widow	316 (13.20)	263 (83.23)	53 (16.77)	10.582	0.001
Married	2078 (86.80)	1859 (89.46)	219 (10.54)		
Residence					
Urban	1689 (70.55)	1490 (88.22)	199 (11.78)	2.729	0.256
Rural	475 (19.84)	431 (90.74)	44 (9.26)		
Rural–urban	230 (9.61)	201 (87.39)	29 (12.61)		
Education level					
College degree and below	1750 (73.10)	1572 (89.83)	178 (10.17)	9.152	0.002
Bachelor’s degree and above	644 (26.90)	550 (85.40)	94 (14.60)		
Personal monthly income					
≤ 3000	879 (36.72)	776 (88.28)	103 (11.72)	1.462	0.691
3001 ~ 6000	1146 (47.87)	1024 (89.35)	122 (10.65)		
6001 ~ 9000	261 (10.90)	227 (86.97)	34 (13.03)		
≥ 9000	108 (4.51)	95 (87.96)	13 (12.04)		
Medical insurance					
Urban resident basic medical insurance	938 (39.18)	834 (88.91)	104 (11.09)	2.667	0.615
Urban Employee basic medical insurance	1386 (57.89)	1229 (88.67)	157 (11.33)		
Commercial health insurance	14 (0.58)	13 (92.86)	1 (7.14)		
Publicly-funded medical care	45 (1.88)	37 (82.22)	8 (17.78)		
None	11 (0.46)	9 (81.82)	2 (18.18)		
Self-rated health status					
Good	2213 (92.44)	1960 (88.57)	253 (11.43)	0.145	0.703
Bad	181 (7.56)	162 (89.50)	19 (10.50)		
Chronic disease					
No	1473 (61.53)	1280 (86.90)	193 (13.10)	11.521	0.001
Yes	921 (38.47)	842 (91.42)	79 (8.58)		
Were you sick in the last 2 weeks?					
Yes	321 (13.41)	283 (66.16)	38 (11.84)	0.084	0.773
No	2073 (86.59)	1839 (88.71)	234 (11.29)		
Walking time to the nearest healthcare center (min)					
< 15	1512 (63.16)	1358 (89.81)	154 (10.19)	5.641	0.018
≥ 15	882 (36.84)	764 (86.62)	118 (13.38)		
The medical institution chosen for the first visit					
Non-primary health service institutions	620 (25.90)	459 (74.03)	161 (25.97)	177.239	< 0.001
Primary health service institutions	1774 (74.10)	1663 (93.74)	111 (6.26)		
Number of visits to primary healthcare institutions in the last year					
< 3	1525 (63.70)	1322 (86.69)	203 (13.31)	15.858	< 0.001
≥ 3	869 (36.30)	800 (92.06)	69 (7.94)		
Awareness of FDCS					
Good	1913 (79.91)	1781 (93.10)	132 (6.90)	294.934	< 0.001
Fair	394 (16.46)	307 (77.92)	87 (22.08)		
Bad	87 (3.63)	34 (39.08)	53 (60.92)		
Mismatch between the services provided by the contracted FDs and the real medical needs					
Yes	579 (24.19)	510 (88.08)	69 (11.92)	184.950	< 0.001
No	1420 (59.31)	1337 (94.15)	83 (5.85)		
Not sure	395 (16.50)	275 (69.62)	120 (30.38)		
Health-seeking behavior change after signing the contract with FDs					
First, go to the FDs in any case of illness and be more willing to go to the primary health service than before	1337 (55.85)	1265 (94.61)	72 (5.39)	192.619	< 0.001
Go to the FDs for minor illnesses and the hospital for serious illness	854 (35.67)	723 (84.66)	131 (15.34)		
Go to the FDs for healthcare services and the hospital for treatment	183 (7.64)	128 (69.95)	55 (30.05)		
First, go to the hospital in any case of illness	20 (0.84)	6 (30.00)	14 (70.00)		
Were you willing to switch FDs?					
Yes	233 (9.73)	204 (87.55)	29 (12.45)	695.83	< 0.001
No	1875 (78.32)	1796 (95.79)	79 (4.21)		
Not sure	286 (11.95)	122 (42.66)	164 (57.34)		
Whether you were satisfied with FDCS?					
No	47 (1.96)	29 (61.70)	18 (38.30)	34.539	< 0.001
Yes	2347 (98.04)	2093 (89.18)	254 (10.82)		
Whether you trusted in FDs?					
No	43 (1.80)	19 (44.19)	24 (55.81)	85.913	< 0.001
Yes	2351 (98.20)	2103 (89.45)	248 (10.55)		

All the variables with a statistical significance (*p* < 0.05) in the univariate Chi-square analysis were included in the mixed-effect logistic regression analysis (Table 2). The results presented in Table 2 indicate that ICC was 4.3%. Residents preferring the primary health service institutions for the first visit (OR = 2.096, 95% CI = 1.460–3.009), having a better knowledge of FDCS (Fair: OR = 0.445, 95% CI = 0.301–0.659; Bad: OR = 0.086, 95% CI = 0.046–0.160), more willing to go to the primary health service in any case of illness after signing the contract with FDs (Go to the FDs for minor illnesses and the hospital for serious illness: OR = 0.587, 95% CI = 0.400–0.861; Go to the FD for healthcare services and the hospital for treatment: OR = 0.350, 95% CI = 0.200–0.612; First go to the hospital in any case of illness: OR = 0.168, 95% CI = 0.042–0.672), not intending to switch FDs (No: OR = 3.003, 95% CI = 1.780–5.065; Not sure: OR = 0.160, 95% CI = 0.093–0.276), satisfied with FDCS (OR = 2.627, 95% CI = 1.062–6.498), and trusting in FDs (OR = 3.432, 95% CI = 1.393–8.454) had significantly a higher level of willingness to maintain contracts with FDs.

**Table 2:** Mixed-effect logistic regression analysis on the influencing factors of residents’ willingness to renew contracts with FDs in China.

Variables	*Β*	*SE*	*Z*	*P*	OR	95% CI
Age (Ref.: 18 ~ 44)							
45 ~ 64	0.269	0.229	1.17	0.241	1.308	0.835–2.050
≥ 65	−0.436	0.286	−1.53	0.127	0.646	0.369–1.131
Marital status (Ref.: Divorced/widow/unmarried)						
Married	0.270	0.237	1.14	0.255	1.311	0.823–2.087
Education level (Ref.: College degree and below)						
Bachelor degree and above	0.085	0.211	0.40	0.687	1.089	0.720–1.460
Chronic disease (Ref.: No)						
Yes	0.251	0.230	1.09	0.276	1.285	0.819–2.016
Walking time to the nearest healthcare center (min) (Ref.: < 15)						
≥ 15	−0.199	0.177	−1.12	0.261	0.819	0.579–1.160
The medical institution chosen for the first visit (Ref.: Non-primary health service institutions)						
Primary health service institutions	0.740	0.185	4.01	< 0.001	2.096	1.460–3.009
Number of visits to primary healthcare institutions in the last year (Ref.: < 3)						
≥ 3	0.144	0.208	0.69	0.490	1.154	0.768–1.736
Awareness of FDCS (Ref.: Good)						
Fair	−0.809	0.200	−4.05	< 0.001	0.445	0.301–0.659
Bad	−2.452	0.316	−7.75	< 0.001	0.086	0.046–0.160
Mismatch between the services provided by the contracted FDs and the real medical needs (Ref.: Yes)						
No	0.050	0.221	0.22	0.822	1.051	0.681–1.621
Not sure	−0.398	0.237	−1.68	0.093	0.672	0.422–1.069
Health-seeking behavior changes after signing the contract with FDs (Ref.: First go to the family doctor in any case of illness and more willing to go to the primary health service than before)						
Go to the FDs for minor illnesses and the hospital for serious illness	−0.533	0.195	−2.73	0.006	0.587	0.400–0.861
Go to the FDs for healthcare services and the hospital for treatment	−1.051	0.285	−3.68	< 0.001	0.350	0.200–0.612
First, go to the hospital in any case of illness	−1.785	0.708	−2.52	0.012	0.168	0.042–0.672
Were you willing to switch FDs (Ref.: Yes)?						
No	1.100	0.267	4.12	< 0.001	3.003	1.780–5.065
Not sure	−1.831	0.278	−6.59	< 0.001	0.160	0.093–0.276
Whether you were satisfied with FDCS (Ref.: No)?						
Yes	0.966	0.462	2.09	0.037	2.627	1.062–6.498
Whether you trusted in FDs (Ref.: No)?						
Yes	1.233	0.460	2.68	0.007	3.432	1.393–8.454

Ref., reference; CI, confidence interval; FDs, family doctors; FDCS, family doctor contract service; OR, odds ratio; min, minutes.

Adjusted for geographical regions.

*χ*^2^ = 403.65; *p* < 0.001; ICC = 4.3%.

### Meta-analysis

**Figure 2:**
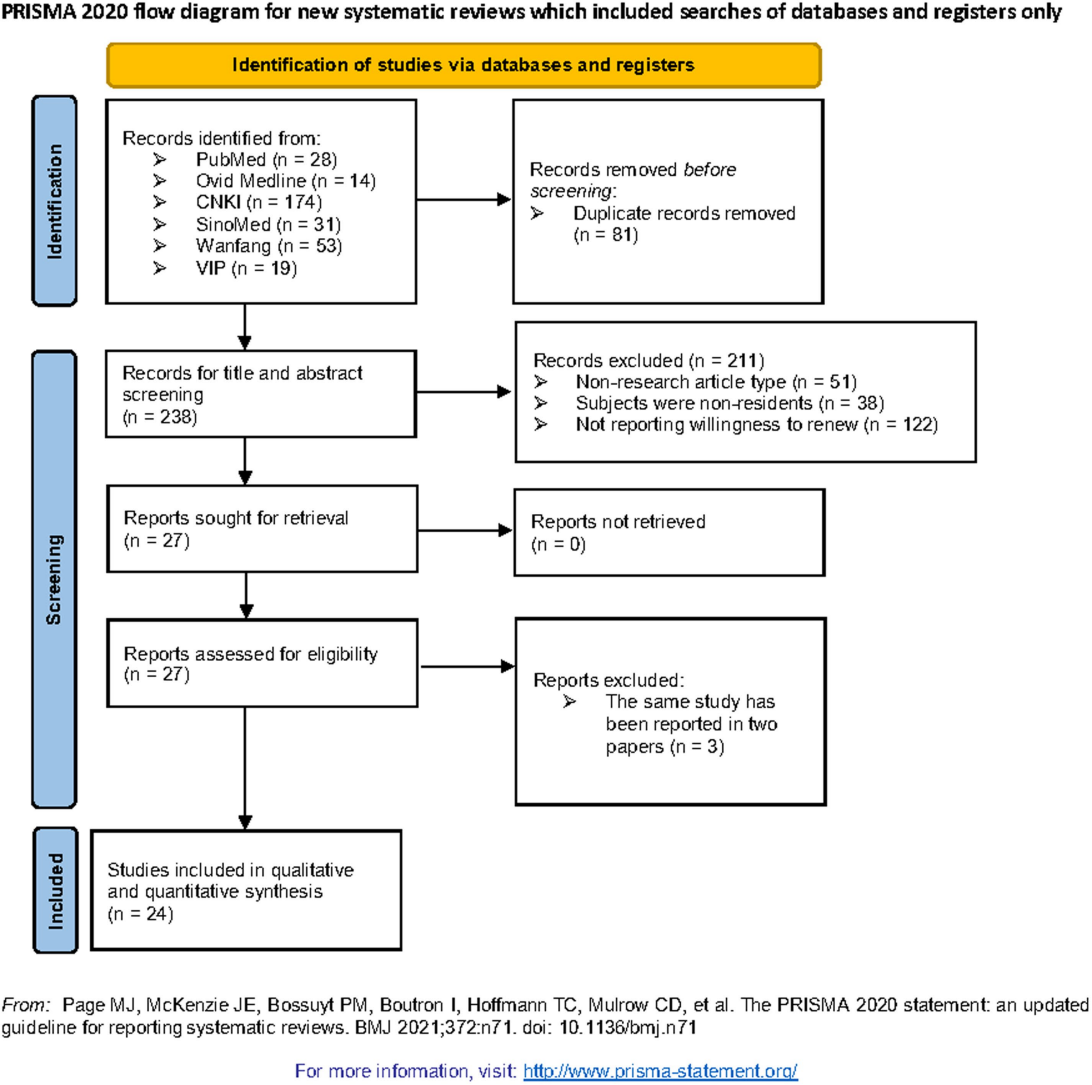
Flow chart of relevant study identification in relation to residents’ willingness to renew contracts with FDs.

The process of study selection and exclusion is illustrated in Figure 2. Initially, 238 articles were screened, and 27 articles were selected for further full-text assessment after the title and abstract screening. Finally, 24 studies were included in this meta-analysis.

**Figure 3:**
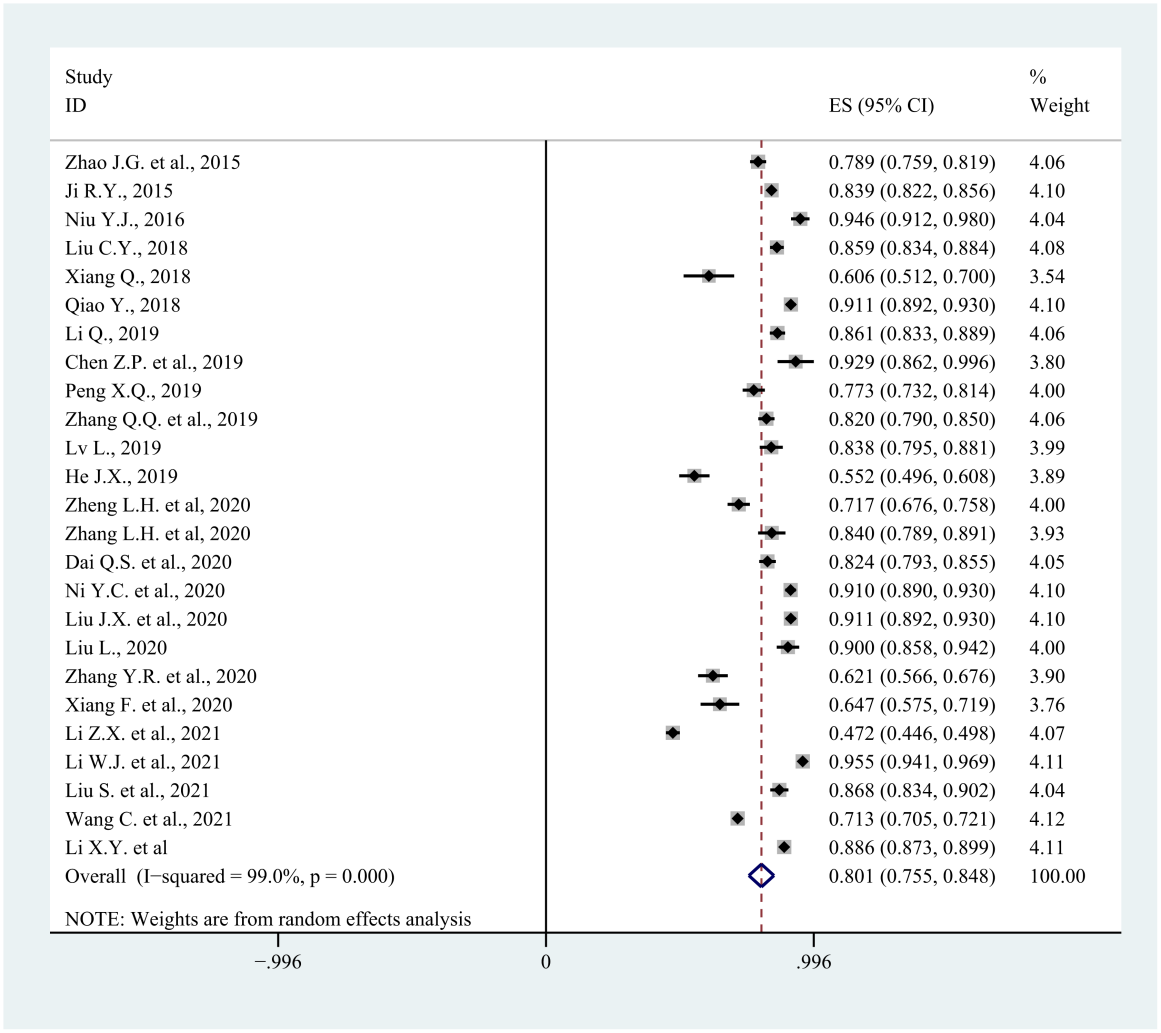
The forest plot of 25 eligible studies in the meta-analysis.

The characteristics of the 25 eligible studies are shown in Supplementary file S3. The sample sizes ranged from 56 to 11250 (median = 568; IQR = 539). The included study quality scores ranged from 2 to 7, with an average score of 4. The pooled proportion of consumers willing to renew their existing contracts with FDs was 80.1% (95% CI: 75.5–84.8%) with significant heterogeneity across studies (*I^2^* = 99.0%, *p* < 0.001) (Figure 3). Being of an older age group, married, having chronic diseases, choosing primary medical institutions for the first contact, having a good knowledge of FDCS or FDs, being satisfied with FDCS and the medical skills of FDs, and trusting in FDs were positively associated with residents’ willingness to renew contracts (Table 3). In the subgroup analysis, search year, medical institution chosen for first visit contact, awareness of FDCS/FDs, satisfaction with FDCS/FDs and with the medical skill of FDs, and trust in FDs were statistically significant associated with the renewal rate (*p* < 0.05) (Table 4). Visual inspection of a funnel plot did not identify substantial asymmetry. Publication bias was not identified in this meta-analysis, with the value of p for the Egger’s test being 0.938.

**Table 3:** Meta-analysis of risk factors associated with residents’ willingness to renew contracts with FDs.^a^

Characteristics	Studies (n)	Pooled OR	95% CI	*I^2^* (%)	*P* for heterogeneity
Age (Ref.: youngest age group)					
Elder age group	5 (22, 27, 28, 35)	1.652	1.076–2.536	71.5	0.007
Marital status (Ref.: spouseless)					
Having a spouse	3 (28, 36)	1.357	1.045–1.763	0.0	0.494
Education level (Ref.: lowest level of education)					
Higher level of education	5 (28, 35, 36, 53)	0.824	0.647–1.048	70.1	0.010
Medical insurance (Ref.: none)					
Have	3 (21, 35, 54)	1.080	0.275–4.246	77.6	0.012
Chronic disease (Ref.: no)					
Yes	4 (16, 27, 35)	2.128	1.367–3.310	61.0	0.053
Walking time to the nearest healthcare center (min) (Ref.: < 15)					
≥15 min	4 (21, 25, 28, 35)	1.134	0.866–1.484	66.6	0.030
Whether you agreed with the “First contact” at community health services (Ref.: no)?					
Yes	8 (14, 16, 17, 22, 28, 35, 54)	2.451	1.238–4.853	92.1	< 0.001
Whether you agreed that the FDCS/FD was necessary (Ref.: no)?					
Yes	3 (14, 16, 22)	2.424	1.741–3.375	0.0	0.845
Awareness of FDCS/FDs (Ref.: bad)					
Good	3 (16, 17)	2.050	1.130–3.718	88.7	< 0.001
Whether you were satisfied with the medical skills of contracted FDs (Ref.: no)?					
Yes	3 (22, 25, 36)	2.951	1.182–7.368	90.0	< 0.001
Whether you were satisfied with FDCS/FDs (Ref.: no)?					
Yes	7 (17, 21, 22, 24, 27, 28)	5.171	1.561–17.133	98.4	< 0.001
Whether you trusted in FDs (Ref.: no)?					
Yes	5 (21, 22, 24, 25)	3.494	2.129–5.734	73.0	0.005

^a^Results were pooled based on our original study and other Chinese studies.

**Table 4:** Subgroup meta-analysis of pooled renewing contract rates in Chinese residents.^a^

Subgroup	Studies	Pooled renewal rate (%) (95% CI)	*P*	*I^2^* (*%*)	*Q*
Search year					
2013–2017	8 (14, 22–24, 26, 35, 54, 55)	87.8 (84.3–91.2)	< 0.001	93.58 99.30	9.03
2018–2020	15 (15–18, 20, 21, 25, 27, 28, 36, 53, 56–58)	75.9 (68.9–82.8)			
Region					
Eastern China	17 (14, 16, 20, 21, 23, 24, 26–28, 35, 36, 53–56, 59)	82.4 (76.0–88.7)	0.289	99.23 99.22 96.61	2.48
Central China	4 (18, 21, 22)	78.1 (65.0–91.2)			
Western China	8 (15, 17, 19, 21, 25, 57, 58)	74.9 (68.2–81.7)			
Sampling method					
Random	20 (14–16, 18, 19, 21–24, 27, 36, 53–59)	78.6 (72.3–84.9)	0.506	99.35 95.39	0.44
Non-random	5 (17, 20, 25, 26, 35)	82.2 (73.7–90.7)			
Quality score					
≤ 4	16 (15, 18, 20, 22–25, 27, 28, 35, 36, 54, 56, 58, 59)	80.3 (73.6–87.1)	0.749	98.91 99.08	0.10
> 4	8 (14, 16, 17, 19, 21, 26, 53, 57)	78.6 (69.9–87.2)			
Age (years old)					
<60	4 (19, 26, 28, 35)	78.8 (52.0–105.5)	0.477	99.39 86.58	0.50
≥60	6 (16, 19, 24, 26, 28, 35)	88.6 (83.7–93.6)			
Marital status					
Unmarried/divorced/widow	11 (15, 17, 19, 21–24, 26, 35, 36)	80.3 (71.1–89.5)	0.932	98.32 99.03	0.01
Married	12 (15, 17, 19, 21–24, 26, 28, 35, 36)	80.8 (73.3–88.2)			
Residence					
Rural	5 (21, 35, 36, 59)	74.3 (57.9–90.7)	0.920	99.25 99.77 99.19	0.17
Urban	4 (21, 35, 36)	74.1 (51.7–96.4)			
Rural–urban	2 (36)	64.4 (19.1–109.7)			
Chronic disease					
No	10 (16, 17, 19, 21, 22, 26, 27, 35, 53)	79.6 (70.8–88.4)	0.161	99.03 97.12	1.96
Yes	11 (16, 17, 19, 21, 22, 24, 26, 27, 35, 53)	87.0 (81.5–92.5)			
Walking time to the nearest healthcare center (min)					
< 15	5 (21, 26, 28, 53)	89.5 (82.3–96.7)	0.270	98.83 97.97	1.22
≥ 15	7 (15, 21, 22, 26, 28, 35)	83.3 (74.9–91.7)			
The medical institution chosen for the first visit					
Non-primary health service institutions	11 (14–17, 19, 22, 26–28, 35)	71.8 (64.0–79.6)	< 0.001	93.82 92.84	17.37
Primary health service institutions	11 (14–17, 19, 22, 26–28, 35)	89.6 (86.5–92.7)			
Awareness of FDCS/FDs					
Good/fair	4 (14–16)	85.3 (80.4–90.2)	0.005	93.61 90.86	7.98
Bad	4 (14–16)	58.4 (40.5–76.4)			
Whether you were satisfied with FDCS/FDs?					
No	10 (17, 18, 21–24, 26–28)	54.5 (40.3–68.7)	< 0.001	96.16 98.22	22.21
Yes	10 (17, 18, 21–24, 26–28)	90.0 (85.9–94.1)			
Whether you were satisfied with the medical skills of FDs?					
No	4 (17, 22, 25, 36)	50.1 (23.3–76.9)	0.023	97.88 96.15	5.20
Yes	4 (17, 22, 25, 36)	82.8 (74.2–91.5)			
Whether you were satisfied with the service attitude of FDs?					
No	3 (22, 25, 36)	52.5 (23.5–81.5)	0.071	97.95 97.99	3.26
Yes	3 (22, 25, 36)	81.7 (68.9–94.4)			
Whether you trusted in FDs?					
No	6 (21–25)	55.3 (35.1–75.5)	0.002	98.99 98.16	9.47
Yes	6 (21–25)	88.0 (83.0–92.9)			
Whether the studies were conducted before COVID-19^b^					
No	3 (36, 57)	82.0 (77.5–86.4)	0.139	99.35	2.19
Yes	22 (14–28, 35, 53–56, 58, 59)	66.9 (47.4–86.4)		98.63	

^a^Results were pooled based on our original study and other Chinese studies.

^b^Researches completed in 2019 and before were considered pre-COVID-19, because the outbreak was in December 2019.

## Discussion

The constituent ratio of residents who were willing to renew contracts with FDs was 88.64%. This percentage was higher than most of the studies of its kind conducted in other Chinese regions as well as the ratio estimated in this meta-analysis (80.1%). We found that residents’ willingness to renew contracts with FDs varied considerably in different studies, suggesting that there may be differences in the implementation effectiveness of FDCS in various regions of China. In addition, the discrepancy may be in response to different study periods, sample sizes, practice settings, and selection.

There was a statistically significant difference in willingness to renew contracts across Eastern (83.48%), Central (91.91%), and Western China (88.25%). This was different from the results of Wang et al. (21) that the renewal rate in Eastern China was significantly higher than that in Central and Western China. One possible reason was the limitation of city selection that only included two major cities in each region. Though it was generally agreed that the asymmetrical allocation of medical resources in China was in a pattern of more from east to west and less from north to south (37), as of 2018, a total of 1,586 FD teams had been organized in Nanning, Western China, with a contract rate of 38.05% (38) for the permanent resident population of 7,254,100 (39). In contrast, there were 1,398 FD teams (40) in Wuhan, Central China, in 2018, with a permanent resident population of 11,081,000 (41), and 1,269 FD teams (42) in Suzhou, Eastern China, in 2019 with a permanent resident population of 10,750,000 (43); a lower population with comparatively more FD teams reflects faster progress of FDCS system in Nanning, which may have contributed to a greater likelihood to renew contracts.

In this study, consumer intention to maintain contracts with FDs increased from 85.64% of residents aged 18–44 years old to 91.50% aged 45–64 and 89.91% over 65, and in the meta-analysis of pooled OR, age was also positive correlative with consumer intention to renew contracts (pooled OR = 1.652, 95% CI = 1.076–2.536). Similarly, having chronic disease was proved to be a significantly positive correlative factor with contract renewal in the univariate analysis (*p* < 0.001) and the meta-analysis (pooled OR = 2.128, 95% CI = 1.367–3.310). One possible reason was that the aged people generally have more concurrent health issues, often in the presence of severe ill health and the need for treatment that serve as the main drivers of their frequent clinic presentations (44), which would motivate them to seek more affordable, convenient, and continuous healthcare services provided by FDs. The same is true for all patients with chronic conditions. Another possible reason was that the older adult and patients with chronic diseases were key service objects of FDCS, and they can receive priority service, door-to-door service, and long-term prescription service (45), which encourages them to maintain the doctor–patient relationship formalized by an FDCS contract. One study suggested that primary medical resources would benefit more residents who needed timely medical treatment, and the allocation of medical resources would also be more reasonable (46). Thus, our results suggest that persisting with improving priority services for key population groups (the older age group and the group with chronic diseases) may help achieve a better renewal rate for the Chinese FDCS and a more rational allocation of health resources.

Good awareness of FDCS or FDs, trusting in FDs, and being satisfied with FDs or FDCS were significantly positively related to consumer intention to renew contracts with FDs, which was proved in the univariate analysis, in the mixed-effect logistic regression analysis, and in the subgroup meta-analysis. In addition, the results in the subgroup meta-analysis implied that, compared with the attitudes of FDs, Chinese residents may be more concerned about the medical skills of particular doctors. The findings suggest that it may be helpful to add propaganda content about the medical achievements and medical practice experience of FDs, rather than focusing solely on the content of FDCS, as is currently the case.

In addition, choosing primary healthcare institutions for an initial consultation significantly positively affected consumer intention to renew their contracts, which was proved in both mixed-effect regression analysis and subgroup meta-analysis. Here the diseases referred to as common and frequent diseases did not include acute and severe conditions. Residents’ satisfaction and trust with FDs were important factors affecting their likelihood to seek the initial consultation at the FDs (47); therefore, this factor reflected residents’ trust and satisfaction with FDs to a certain extent. Another possible reason was that consumers who chose primary healthcare institutions for an initial consultation were more likely to use primary healthcare services, thereby patients established a long-term friendly relationship with FDs and thus were more willing to maintain their contracts (48).

Intriguingly, we included the item “health-seeking behavior change after signing the contract with FDs,” which was not included in any other study, and “choose to go to the FD in any case of illness and more willing to go to the primary health service” had a significantly positive relation with residents’ willingness to renew contracts. Results in the mixed-regression analysis suggested that residents who preferred to seek initial help at the hospital in any case of illness, even though they had been in contract with FDs, would show a low willingness to maintain contracts with FDs, indicating that signing the contract with FDs is only the very starting point of FDCS, that we should help residents to use the medical services provided by FD team more frequently and cultivate their habit of using primary medical services. However, the proportion of contracted residents using contracted medical services and health management services was fairly low. For example, approximately half of the residents did not utilize the service within one contract period in the Pearl River delta in southern China, leading to a rise in the withdrawal from the FDCS system due to a lack of no need for and knowledge of family doctor services (49). We therefore suggest using the renewal rate as an indicator of user stickiness and activeness and service quality in the long term.

Finally, the results in the subgroup meta-analysis showed that the pooled renewing contract rates of studies conducted before and after the COVID-19 outbreak were 82.4% (95% CI: 77.7–87.0%) and 66.9% (95% CI: 35.3–98.4%), respectively. Moreover, the difference in renewing contract rates between the two subgroups was not statistically significant (*p* > 0.05). This may be due to the insufficient number of included studies conducted after the COVID-19 outbreak (3 studies, including our research). To date, a few studies have shown that during the COVID-19 pandemic, the doctor–patient relationship in China has been steadily improving (50). Positive media coverage of medical staff, free online consultations, a psychiatric hotline, and free treatment for confirmed and suspected COVID-19 patients had a significant positive impact on doctor–patient relationships (51). Patients had high levels of trust in doctors (52). These factors may help increase the willingness of residents to renew their FDCS. Additional studies investigating the effect of COVID-19 on willingness to maintain contracts with FDs are warranted.

### Strengths and limitations

To the best of our knowledge, the present study is the first to conduct a national cross-section survey combined with meta-analysis to comprehensively summarize the willingness to maintain contracts with FDs among Chinese residents and potential influencing factors.

Nevertheless, there were some limitations in this study. First, sampling was applied in this study, choosing only six cities in China, which could not fully reflect residents’ willingness to renew their contracts with FDs in China. Second, there may be other factors affecting residents’ willingness to maintain contracts with FDs, such as the reimbursement ratio of medical insurance, service contents and the payment of FDCS, the development of follow-up services, and health expenditure. Third, there may be a self-reporting bias in the study. Finally, a high level of heterogeneity was observed in this study, as expected when pooling estimates across time and locations. The heterogeneity across studies may result from differences in samples and practice settings. However, consistent results from various subgroup analyses indicated that our findings were relatively reliable and robust, and the heterogeneity can be overestimated when studies with large sample sizes are pooled (60).

## Conclusion

Consumer willingness to renew contracts with FDs varied considerably from region to region in China. The strategy of giving priority services to priority population groups in the FDCS system was successful in China, helping to increase the renewal rate and expand the coverage of FDCS. To increase consumer likelihood of contract renewal, it is important to publicly promote the benefits of FDCS from a consumer perspective, and enhance belief, satisfaction, and trust in FDCS and FDs. It could be argued that the renewal rate could become an indicator to assess consumer levels of morbidity sickness and the resultant service quality of the FDCS in the long term.

## Data availability statement

The raw data supporting the conclusions of this article will be made available by the authors, without undue reservation.

## Ethics statement

The studies involving humans were approved by the Ethics Committee of the Wenzhou Medical University Institutional Review Board, Wenzhou, China (no. 2021–019). The studies were conducted in accordance with the local legislation and institutional requirements. The participants provided their written informed consent to participate in this study.

## Author contributions

XL, JY, and YG conceived and designed the study. XL, JF, GQ, ZL, and YZ participated in the acquisition of data. XL analyzed the data and wrote the draft of the manuscript. JY, QC, AF, HJ, and YG advised on methodology. YG is the guarantor of this study has full access to all the data in the study and took responsibility for its integrity and the accuracy of the data analysis. All authors contributed to writing, reviewing, or revising the manuscript, read, and approved the final version.
