# Supplementary material for: Willingness to maintain contracts with family doctors among Chinese residents: results from one national cross-sectional study and a meta-analysis of 25 studies
Source: Front Public Health. 2023 Dec 22;11:1162824. doi: 10.3389/fpubh.2023.1162824 (PMC10770837; doi:10.3389/fpubh.2023.1162824)
Supplement: Supplementary file 1 [file Table_1.DOCX]

| **Descriptive statistics and univariate analysis of the differences in associated with willingness to maintain contracts with FDs among eastern, central and western China** | | | | | | | | | | | |
| --- | --- | --- | --- | --- | --- | --- | --- | --- | --- | --- | --- |
| **Variables** | **Eastern China** | | | **Central China** | | | **Western China** | | | ***χ^2^*** | ***P*** |
|  | **Total (%)** | **Willing (%)** | **Unwilling (%)** | **Total**  **(%)** | **Willing (%)** | **Unwilling (%)** | **Total (%)** | **Willing (%)** | **Unwilling (%)** |  |  |
| **Age (years old)** | | | | | | | | | | | |
| 18~44 | 270 (46.47) | 221 (81.85) | 49  (18.15) | 329 (32.48) | 290 (88.15) | 39  (11.85) | 425 (53.13) | 366 (86.12) | 59  (13.88) | 410.496 | < 0.001 |
| 45~64 | 198 (34.08) | 172 (86.87) | 26  (13.13) | 345 (34.06) | 325 (94.20) | 20  (5.80) | 292 (36.50) | 267 (91.44) | 25  (8.56) |  |  |
| ≥65 | 113 (19.45) | 92 (81.42) | 21  (18.58) | 339 (33.46) | 316 (93.22) | 23  (6.78) | 83 (10.38) | 73  (87.95) | 10  (12.05) |  |  |
| **Marital status** | | | | | | | | | | | |
| Unmarried/divorced/widow | 69  (11.88) | 49  (71.01) | 20  (28.99) | 117  (11.55) | 106 (90.60) | 11  (9.40) | 130 (16.25) | 108  (83.08) | 22  (16.92) | 9.792 | 0.007 |
| Married | 512 (88.12) | 436 (85.16) | 76  (14.84) | 896 (88.45) | 825 (92.08) | 71  (7.92) | 670 (83.75) | 598 (89.25) | 72  (10.75) |  |  |
| **Education level** | | | | | | | | | | | |
| College degree and below | 370 (63.68) | 309 (83.51) | 61  (16.49) | 791 (78.08) | 737 (93.17) | 54  (6.83) | 589 (73.63) | 526 (89.30) | 63  (10.70) | 39.113 | < 0.001 |
| Bachelor degree and above | 211 (36.32) | 176 (83.41) | 35  (16.59) | 222 (21.92) | 194 (87.39) | 28  (12.61) | 211 (26.38) | 180 (85.31) | 31  (14.69) |  |  |
| **Chronic disease** | | | | | | | | | | | |
| Yes | 197 (33.91) | 173 (87.82) | 24  (12.18) | 551 (54.39) | 508 (92.20) | 43  (7.80) | 173 (21.63) | 161 (93.06) | 12  (6.94) | 209.516 | < 0.001 |
| No | 384 (66.09) | 312 (81.25) | 72  (18.75) | 462 (45.61) | 423 (91.56) | 39  (8.44) | 627 (78.37) | 545 (86.92) | 82  (13.08) |  |  |
| **Walking time to the nearest healthcare center (min)** | | | | | | | | | | | |
| <15 | 411  (70.74) | 347 (84.43) | 64  (15.57) | 625  (61.70) | 584 (93.44) | 41  (6.56) | 476 (59.50) | 427 (89.71) | 49  (10.29) | 19.883 | < 0.001 |
| ≥15 | 170  (29.26) | 138 (81.18) | 32  (18.82) | 388 (38.30) | 347 (89.43) | 41  (10.57) | 324 (40.50) | 279  (86.11) | 45  (13.98) |  |  |
| **The medical institution chosen for the first visit** | | | | | | | | | | | |
| Non-primary health service institutions | 194 (33.39) | 132 (68.04) | 62  (31.96) | 229 (22.61) | 183 (79.91) | 46  (20.09) | 197 (24.63) | 144 (73.10) | 53  (26.90) | 23.392 | < 0.001 |
| Primary health service institutions | 387 (66.61) | 353 (91.21) | 34  (8.79) | 784 (77.39) | 748 (95.41) | 36  (4.59) | 603 (75.38) | 562 (93.20) | 41  (6.80) |  |  |
| **Number of visits to primary healthcare institutions in the last year** | | | | | | | | | | | |
| <3 | 311 (53.53) | 240 (77.17) | 71  (22.83) | 627 (61.90) | 572 (91.23) | 55  (8.77) | 587 (73.38) | 510 (86.88) | 77  (13.12) | 59.809 | < 0.001 |
| ≥3 | 270 (46.47) | 245 (90.74) | 25  (9.26) | 386 (38.10) | 359 (93.01) | 27  (6.99) | 213 (26.63) | 196 (92.02) | 17  (7.98) |  |  |
| **Awareness of FDCS** | | | | | | | | | | | |
| Good | 465 (80.03) | 410 (88.17) | 55  (11.83) | 777 (76.70) | 746 (96.01) | 31  (3.99) | 671 (83.88) | 625 (93.14) | 46  (6.86) | 18.680 | 0.001 |
| Fair | 91  (15.66) | 67 (73.63) | 24  (26.37) | 202 (19.94) | 166 (82.18) | 36  (17.82) | 101 (12.63) | 74  (73.27) | 27  (26.73) |  |  |
| Bad | 25  (4.30) | 8 (32.00) | 17  (68.00) | 34  (3.36) | 19 (55.88) | 15  (44.12) | 28 (3.50) | 7  (25.00) | 21  (75.00) |  |  |
| **Mismatch between the services provided by the contracted FDs and the real medical needs** | | | | | | | | | | | |
| Yes | 176 (30.29) | 150 (85.23) | 26  (14.77) | 204 (20.14) | 185 (90.69) | 19  (9.31) | 199 (24.88) | 175 (87.94) | 24  (12.06) | 30.845 | < 0.001 |
| No | 295 (50.77) | 266 (90.17) | 29  (9.83) | 654 (64.56) | 625 (95.57) | 29  (4.43) | 471 (58.88) | 446 (94.69) | 25  (5.31) |  |  |
| Not sure | 110 (18.93) | 69 (62.73) | 41  (37.27) | 155 (15.30) | 121 (78.06) | 34  (21.94) | 130 (16.25) | 85  (65.38) | 45  (34.62) |  |  |
| **Health seeking behavior change after signing the contract with FDs** | | | | | | | | | | | |
| First go to the family doctor in any case of illness and more willing to go to the primary health service than before | 360 (61.96) | 327 (90.83) | 33  (9.17) | 541 (53.41) | 527 (97.41) | 14  (2.59) | 436 (54.50) | 411 (94.27) | 25  (5.73) | 18.180 | 0.006 |
| Go to the family doctor for minor illnesses and the hospital for serious illness | 183 (31.50) | 135 (73.77) | 48  (26.23) | 381 (37.61) | 341 (89.50) | 40  (10.50) | 290 (36.25) | 247 (85.17) | 43  (14.83) |  |  |
| Go to the family doctor for healthcare services and the hospital for treatment | 36  (6.20) | 23 (63.89) | 13  (36.11) | 85  (8.39) | 61 (71.76) | 24  (28.24) | 62 (7.75) | 44  (70.97) | 18  (29.03) |  |  |
| First go to the hospital in any case of illness | 2  (0.34) | 0  (0.00) | 2  (100.00) | 6  (0.59) | 2  (33.33) | 4  (66.67) | 12 (1.50) | 4  (33.33) | 8  (66.67) |  |  |
| **Were you willing to** **switch FDs** | | | | | | | | | | | |
| Yes | 77  (13.25) | 66 (85.71) | 11  (14.29) | 73  (7.21) | 68 (93.15) | 5  (6.85) | 83 (10.38) | 70  (84.34) | 13  (15.66) | 31.257 | < 0.001 |
| No | 426 (73.32) | 398 (93.43) | 28  (6.57) | 846 (83.51) | 813 (96.10) | 33  (3.90) | 603 (75.38) | 585 (97.01) | 18  (2.99) |  |  |
| Not sure | 78  (13.43) | 21 (26.92) | 57  (73.08) | 94  (9.28) | 50 (53.19) | 44  (46.81) | 114 (14.25) | 51  (44.74) | 63  (55.26) |  |  |
| **Whether you were satisfied with FDCS** | | | | | | | | | | | |
| Yes | 574 (98.80) | 481 (83.80) | 93  (16.20) | 999 (98.62) | 922 (92.29) | 77  (7.71) | 774 (96.75) | 690 (89.15) | 84  (10.85) | 10.396 | 0.006 |
| No | 7  (1.20) | 4 (57.14) | 3  (42.86) | 14  (1.38) | 9  (64.29) | 5  (35.71) | 26 (3.25) | 16  (61.54) | 10  (38.46) |  |  |
| **Whether you were trusted in FDs** | | | | | | | | | | | |
| Yes | 577 (99.31) | 485 (84.06) | 92  (15.94) | 996 (98.32) | 922 (92.57) | 74  (7.43) | 778 (97.25) | 696 (89.46) | 82  (10.54) | 8.248 | 0.016 |
| No | 4  (0.69) | 0  (0.00) | 4  (100.00) | 17  (1.68) | 9  (52.94) | 8  (47.06) | 22 (2.75) | 10  (45.45) | 12  (54.55) |  |  |
