# Supplementary material for: Willingness to maintain contracts with family doctors among Chinese residents: results from one national cross-sectional study and a meta-analysis of 25 studies
Source: Front Public Health. 2023 Dec 22;11:1162824. doi: 10.3389/fpubh.2023.1162824 (PMC10770837; doi:10.3389/fpubh.2023.1162824)
Supplement: Supplementary file 2 [file Table_2.DOCX]

**Search Strategy**

*CNKI Search strategy*

1. TI=‘family doctor’ + ‘family doctor service’ + ‘family doctor system’ + ‘family doctor contract service’
2. KY=% ‘family doctor’ + % ‘family doctor service’ + % ‘family doctor system’ + % ‘family doctor contract service’
3. 1 OR 2
4. KY=% ‘willingness to renew’ + % ‘renewal’ + % ‘willingness to extend’ + % ‘extension’
5. FT=‘willingness to renew’ + ‘willingness to extend’
6. 4 OR 5
7. 3 AND 6

*Wanfang Search strategy*

1. Full text: "willingness to renew" or "willingness to extend"
2. Title/abstract: willingness to renew or renew or willingness to extend or extend
3. 1 or 2
4. Title/abstract: family doctor or family doctor service or family doctor system or family doctor contract service
5. 3 and 4

*VIP Search strategy*

1. M=willingness to renew OR renew OR willingness to extend OR extend
2. U="willingness to renew" OR "willingness to extend"
3. 1 OR 2
4. M= family doctor OR family doctor service OR family doctor system OR family doctor contract service
5. 3 AND 4

*SinoMed Search strategy*

1. "physician, family"[Exp]
2. "renew"[Abstract] OR "willingness to renew"[Abstract] OR "willingness to extend"[Abstract] OR "extend"[Abstract]
3. 1 AND 2

*PubMed Search strategy*

1. health services[MeSH Terms]
2. contract services[MeSH Terms]
3. 1 OR 2
4. family physician*[Title/Abstract]
5. family doctor*[Title/Abstract]
6. general physician*[Title/Abstract]
7. general practitioner*[Title/Abstract]
8. 4 OR 5 OR 5 OR 7
9. renew*[Title/Abstract]
10. maintain*[Title/Abstract]
11. continu*[Title/Abstract]
12. exten*[Title/Abstract]
13. 9 OR 10 OR 11 OR12
14. China[Title/Abstract]
15. Chinese[Title/Abstract]
16. 14 OR 15
17. 3 AND 8 And 13 AND 16

*Ovid Medline Search strategy*

1. *Contract Services/ or *Primary Health Care/ or *Health Services/
2. (family physician* or family doctor* or general physician* or general practitioner*).ab,ti.
3. (renew* or maintain* or continu* or exten*).ab,ti.
4. (China or Chinese).ab,ti.
5. 1 and 2 and 3 and 4
