# Supplementary material for: Willingness to maintain contracts with family doctors among Chinese residents: results from one national cross-sectional study and a meta-analysis of 25 studies
Source: Front Public Health. 2023 Dec 22;11:1162824. doi: 10.3389/fpubh.2023.1162824 (PMC10770837; doi:10.3389/fpubh.2023.1162824)
Supplement: Supplementary file 3 [file Table_3.DOCX]

| **Supplementary Table 2 Characteristics of 25 eligible studies included in meta-analysis** | | | | | | | | | |
| --- | --- | --- | --- | --- | --- | --- | --- | --- | --- |
| Study | Search Year | Provinces | Region | Sample size | Willing to Renew | Rate | Effective Response  Rate (%) | Sampling Method | Quality Score |
| Zhao J.G. et al., 2015 | 2013 | Beijing | Eastern | 721 | 569 | 0.789 | 95.37 | Random | 5 |
| Ji R.Y., 2015 | 2014 | Beijing | Eastern | 1876 | 1574 | 0.839 | 94.17 | Stratified random | 4 |
| Niu Y.J., 2016 | 2015 | Guangdong | Eastern | 167 | 158 | 0.946 | 97.41 | Convenience | 5 |
| Liu C.Y., 2018 | 2017 | Hubei | Central | 735 | 631 | 0.859 | 98.00 | Random | 4 |
| Xiang Q., 2018 | - | Shanxi | Western | 104 | 63 | 0.606 | 80.75 | Stratified random | 5 |
| Qiao Y., 2018 | 2017 | Guangdong | Eastern | 900 | 820 | 0.911 | 90.00 | Simple random | 4 |
| Li Q., 2019 | 2018 | Zhejiang | Eastern | 568 | 489 | 0.861 | 94.67 | Stratified random | 3 |
| Chen Z.P. et al., 2019 | - | Shandong | Eastern | 56 | 52 | 0.929 | 99.17 | Multistage stratified random | 2 |
| Peng X.Q., 2019 | 2018 | Chongqing | Western | 397 | 307 | 0.773 | 95.04 | Convenience | 5 |
| Zhang Q.Q. et al., 2019 | 2018 | Gansu | Western | 622 | 510 | 0.820 | 95.69 | Stratified random | 4 |
| Lv L., 2019 | 2019 | Fujian | Eastern, | 284 | 219 | 0.838 | 77.11 | Convenience | 4 |
| He J.X., 2019 | 2018 | Fujian | Eastern | 299 | 165 | 0.552 | 89.79 | Multistage stratified random | 3 |
| Zheng L.H. et al, 2020 | 2019 | Ningxia | Western | 456 | 327 | 0.717 | 95.00 | - | 4 |
| Zhang L.H. et al, 2020 | 2019 | Ningxia | Western | 200 | 168 | 0.840 | 86.96 | Random | 3 |
| Dai Q.S. et al., 2020 | 2019 | Zhejiang | Eastern | 590 | 486 | 0.824 | 90.77 | Random | 5 |
| Ni Y.C. et al., 2020 | 2019 | Zhejiang | Eastern | 819 | 745 | 0.910 | 98.67 | Stratified random cluster | 4 |
| Liu J.X. et al., 2020 | 2016 | Guangdong | Eastern | 900 | 820 | 0.911 | 90.00 | Multistage stratified random | 4 |
| Liu L., 2020 | 2016 | Guangdong | Eastern | 200 | 180 | 0.900 | 86.96 | - | 4 |
| Zhang Y.R. et al., 2020 | 2019 | Shanxi | Central | 298 | 185 | 0.621 | 99.33 | Multistage & random | 4 |
| Xiang F. et al., 2020 | 2020 | Sichuan | Western | 170 | 110 | 0.647 | 96.86 | Stratified random | 6 |
| Li Z.X. et al., 2021 | 2020 | Shandong | Eastern | 1445 | 682 | 0.472 | 96.33 | Multistage stratified random | 3 |
| Li W.J. et al., 2021 | 2018 | Shandong | Eastern | 823 | 786 | 0.955 | 72.24 | Multistage stratified cluster | 5 |
| Liu S. et al., 2021 | 2017 | Jiangsu | Eastern | 385 | 334 | 0.868 | 100.00 | Stratified random cluster | 2 |
| Wang C. et al., 2021 | 2019 | 31 provinces | - | 11250 | 8016 | 0.713 | 96.90 | Multistage stratified random | 7 |
| Li X.Y. et al.^*^ | 2021 | 6 provinces | - | 2394 | 2122 | 0.886 | 97.74 | Multistage stratified random | 7 |
| -: unreported or unsuitable.  *The study conducted by us. | | | | | | | | | |
